# Perivascular Accumulation of β-Sheet-Rich Proteins in Offspring Brain following Maternal Exposure to Carbon Black Nanoparticles

**DOI:** 10.3389/fncel.2017.00092

**Published:** 2017-03-31

**Authors:** Atsuto Onoda, Takayasu Kawasaki, Koichi Tsukiyama, Ken Takeda, Masakazu Umezawa

**Affiliations:** ^1^Department of Hygienic Chemistry, Graduate School of Pharmaceutical Sciences, Tokyo University of ScienceNoda, Japan; ^2^The Center for Environmental Health Science for the Next Generation, Research Institute for Science and Technology, Organization for Research Advancement, Tokyo University of ScienceNoda, Japan; ^3^Research Fellow of Japan Society for the Promotion of ScienceTokyo, Japan; ^4^Infrared Free Electron Laser Research Center, Research Institute for Science and Technology, Organization for Research Advancement, Tokyo University of ScienceNoda, Japan; ^5^Department of Chemistry, Faculty of Science, Tokyo University of ScienceTokyo, Japan; ^6^Department of Materials Science and Technology, Faculty of Industrial Science and Technology, Tokyo University of ScienceTokyo, Japan

**Keywords:** infrared microspectroscopy, protein secondary structure, astrocyte, perivascular macrophage, glial fibrillary acidic protein, aquaporine-4, developmental neurotoxicity, waste clearance

## Abstract

Environmental stimulation during brain development is an important risk factor for the development of neurodegenerative disease. Clinical evidence indicates that prenatal exposure to particulate air pollutants leads to diffuse damage to the neurovascular unit in the developing brain and accelerates neurodegeneration. Maternal exposure to carbon black nanoparticles (CB-NPs), used as a model for particulate air pollution, induces long-lasting diffuse perivascular abnormalities. We aimed to comprehensively characterize the perivascular abnormalities related to maternal NPs exposure using Fourier transform infrared microspectroscopy (*in situ* FT-IR) and classical staining analysis. Pregnant ICR mice were intranasally treated with a CB-NPs suspension (95 μg/kg at a time) on gestational days 5 and 9. Brains were collected 6 weeks after birth and sliced to prepare 10-μm-thick serial sections. Reflective spectra of *in situ* FT-IR were acquired using lattice measurements (*x*-axis: 7, *y*-axis: 7, 30-μm apertures) around a centered blood vessel. We also performed mapping analysis of protein secondary structures. Serial sections were stained with using periodic acid-Schiff or immunofluorescence to examine the phenotypes of the perivascular areas. Peaks of amide I bands in spectra from perivascular areas were shifted by maternal NPs exposure. However, there were two types of peak-shift in one mouse in the exposure group. Some vessels had a large peak-shift and others had a small peak-shift. *In situ* FT-IR combined with traditional staining revealed that the large peak-shift was induced around blood vessel adjacent to astrocytes with glial fibrillary acidic protein and aquaporin-4 over-expression and perivascular macrophages (PVMs) with enlarged lysosome granules. Furthermore, protein secondary structural analysis indicated that maternal NPs exposure led to increases in β-sheet content and decreases in α-helix content in areas that are mostly close to the centered blood vessel displaying histopathological changes. These results suggest that β-sheet-rich waste proteins, which are denatured by maternal NPs exposure, likely accumulate in the perivascular space as they are processed by the clearance systems in the brain. This may in turn lead the denaturation of PVMs and astrocyte activation. The risk of neurodegeneration may be enhanced by exposure to particulate air pollutants during brain development following the perivascular accumulation of β-sheet-rich waste proteins.

## Introduction

Establishing medical treatments for and preventing central nervous system (CNS) diseases is widely recognized as one of the most challenging health issues in the 21st century worldwide (WHO, 2006). It is known that CNS diseases have multifactorial etiologies, which include endogenous factors (such as gene predisposition and aging) and exogenous factors (repeated and cumulative environmental risk factors). Environmental stimulation during brain development is one of the most important risk factors for CNS diseases, including neurodegenerative disease (Knuesel et al., 2014). Clinical cohort studies have shown clear epidemiological associations between maternal exposure to environmental stimulation and greater risks for schizophrenia, autism, and epilepsy in offspring (Atladóttir et al., 2010; Sun et al., 2011; Brown, 2012). In addition, emerging evidence suggests that prenatal and neonatal infection predisposes individuals to cerebral palsy and age-associated neurodegenerative diseases, such as Alzheimer’s disease (AD) and Parkinson’s disease (PD) generally accompanying accumulation of abnormal structured β-sheet-rich proteins including amyloid-β and proteins comprising Lewy bodies via maternal immune activation (Knuesel et al., 2014; Chin-Chan et al., 2015). Therefore, research on abnormal brain development following prenatal and neonatal stimulation by environmental risk factors will contribute to the understanding of mechanisms underlying the development of diverse CNS diseases and to the establishment of medical treatments and prevention strategies for these diseases.

Clinical evidence indicates that exposure to particulate air pollutants, such as fine (PM_2.5_) and ultrafine particles, is linked to the development of CNS disease (Calderón-Garcidueñas et al., 2014, 2016a; Heusinkveld et al., 2016). Clear associations between prenatal exposure to particulate air pollution, including black carbon, and increased risk of developmental brain disorders, such as autism spectrum disorder and schizophrenia, in offspring has also been demonstrated (Allen et al., 2015; Raz et al., 2015; Woodward et al., 2015). Recent clinical evidence suggests that perinatal exposure to PM_2.5_ leads to diffuse damage to the neurovascular unit, which is composed of endothelial cells, perivascular cells including pericytes, glial cells such as astrocytes, and neurons, in the developing brain. In addition, perinatal exposure to PM_2.5_ may accelerate AD and PD pathology (Calderón-Garcidueñas et al., 2013, 2016a,b; Heusinkveld et al., 2016). In agreement with the above clinical evidence, previous animal studies have demonstrated that maternal exposure to carbon black nanoparticles (CB-NPs), which is used as a model particle for air pollution, induces long-lasting diffuse perivascular abnormalities, including astrogliosis with swollen astrocytic end-feet and histopathological changes in perivascular macrophages (PVMs) (Onoda et al., 2014, 2017). In particular, glial fibrillary acidic protein (GFAP) and aquaporin-4 (Aqp4), which are astrocyte activation markers (Pekny and Nilsson, 2005; Badaut et al., 2012; Haj-Yasein et al., 2011), were dose-dependently upregulated by maternal CB-NP exposure (Onoda et al., 2017). However, the mechanisms underlying the induction of perivascular abnormalities by maternal CB-NPs exposure are still unknown. Comprehensive characterization of the area surrounding brain blood vessels and the histopathological changes in the surrounding tissue are required to elucidate these mechanisms. In addition, detailed characterization of the changes in the neurovascular areas that are induced by NPs exposure during brain development will provide unprecedented knowledge that may be used to help patients with CNS disease. Therefore, the goal of the present study was to characterize the perivascular abnormalities induced by CB-NPs exposure during brain development.

We used Fourier transform infrared microspectroscopy (*in situ* FT-IR) to investigate changes in the neurovascular areas of offspring induced by maternal NPs exposure. Almost all chemical bonds in biomolecules have individual vibrational modes in the IR region. *In situ* FT-IR can be used to elucidate the chemical and structural characteristics of biomolecules, such as proteins, lipids, polysaccharides, and nucleic acids, in regions of interest on tissue sections without the use of contrast agents (Caine et al., 2012; Kawasaki et al., 2016a,b). In fact, *in situ* FT-IR has already been used to analyze the structures of biomolecules and pathology in various cancers (Caine et al., 2012), myocardial infarction (Yang et al., 2011), chronic obstructive pulmonary disease (Whiteman et al., 2008), and neurodegenerative diseases (Choo et al., 1995). These studies demonstrated that *in situ* FT-IR can be used for rapid imaging and characterization of disease progression in vast areas of tissues. Furthermore, *in situ* FT-IR enables the quantitative analysis of biomolecules associated with injury or disease, as the intensities of the corresponding absorption bands in the IR spectrum can be calculated (Zhang et al., 2015). This technique can also analyze the changes in the distribution of protein secondary structure components, for example, in cultured cells with rhodopsin protein mutation (Miller et al., 2015). Classical histopathological analysis techniques, including immunohistochemistry and specific staining, can generally provide the data regarding the distributions of target biomolecules. However, they are not ideal for the quantitative investigation of the expression levels of the target molecule. Although western blotting and enzyme-linked immunosorbent assays can provide quantitative data regarding target protein expression, they are unable to provide us with information regarding the localization of the expression of the target protein in tissues. Proteomics and metabolomics are the comprehensive analysis techniques, but are difficult to apply to small dissected samples, such as the perivascular areas in the brain. Unlike these classical methods, *in situ* FT-IR can be used for quantitative assessment of the molecular and structural compositions of biological tissue sections without the use of additional chemical reagents. Therefore, this technique has the added benefit of reducing the possibility of introducing artifacts. *In situ* FT-IR can thus be used to quantitatively characterize perivascular abnormalities induced by CB-NPs exposure during brain development. The purpose of the present study was to investigate histopathological changes around brain blood vessels that were related to maternal CB-NPs exposure *in situ* using FT-IR microspectroscopy in combination with traditional staining methods.

## Materials and Methods

### Particle

Printex 90, purchased from Degussa Ltd (Frankfurt, Germany), was used as CB-NPs. The manufacturer reported an average primary particle size of 14 nm with a specific surface area of 295–338 m^2^/g and an organic impurity content of less than 1% (>99 carbon wt%, 0.82 nitrogen wt%, and 0.01 hydrogen wt%) (Jacobsen et al., 2007; Jackson et al., 2012). CB-NPs suspension was prepared by using ultra-pure water as previously described (Onoda et al., 2014). The prepared CB-NPs suspension was maintained on ice and used for intranasal instillation within an hour. The data of dynamic light scattering showed that filtered Printex 90 suspension had small agglomerated particles with a peak size of 84.2 nm and polydispersity index of 0.143 (Onoda et al., 2014). This 84.2 nm size corresponds well with the typical small agglomerate sizes observed in Printex 90 samples using transmission electron microscopy (Onoda et al., 2014). The CB-NPs concentration in the suspension was calculated to be 95 μg/mL (Onoda et al., 2014). The filtered Printex 90 was stably suspended in the ultra-pure water. The size distribution of the suspension was not altered until using for the intranasal instillation.

### Animals and Treatments

**FIGURE 1:**
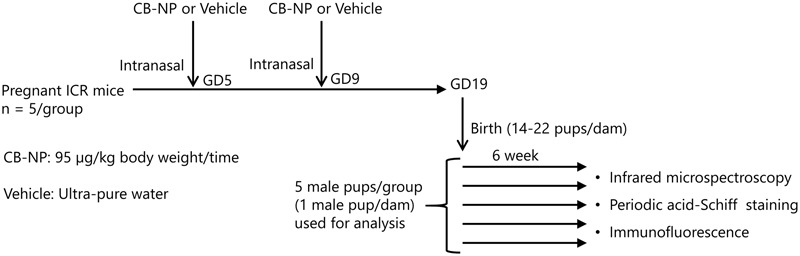
**Summarized schematic for the animal treatments and sample collection**.

Pregnant ICR mice (11 weeks of age) were purchased from SLC Inc. (Hamamatsu, Shizuoka, Japan) and were randomly divided into two groups: CB-NP-exposed (*n* = 5), and control (*n* = 5). The mice were housed under controlled temperature (23 ± 1°C) and humidity (55% ± 5%) with a 12-h dark/light cycle and *ad libitum* access to food and water. Pregnant mice were anesthetized in a box filled with halothane and then taken out from the box when they started to fall asleep. The mice were immediately laid on their backs and treated with 1 mL/kg (body weight) of CB-NPs suspension (95 μg/mL, exposure group) or distilled water (control group) by intranasal instillation into both nostrils. The number of pups per dam was adjusted randomly to 11 or 12 on postnatal day 1. After weaning at 3 weeks of age, 4–6 male offspring per dam were randomly selected and used for analysis. Brains were collected from male offspring 6 and 12 weeks after birth (**Figure 1**). All experiments were performed in accordance with Animal Research: Reporting *In Vivo* Experiments guidelines for the care and use of laboratory animals (Kilkenny et al., 2011) and were approved by Tokyo University of Science’s Institutional Animal Care and Use Committee (Approval Number: Y16057). All sampling was performed under sodium pentobarbital (50 mg/kg) anesthesia, and all efforts were made to minimize suffering.

### Preparation of Serial Sections

Collected brain samples were sliced into coronal sections at 1 mm intervals using a brain blocker. Anesthetized mice were transcardially perfusion-fixed using 4% paraformaldehyde (PFA) in 0.1 M phosphate buffer after perfusion with phosphate buffered saline (PBS). One-mm coronal brain sections were post-fixed in 4% PFA in 0.1 M phosphate buffer for 5 h and cryoprotected in phosphate-buffered sucrose solutions (10% sucrose, 4–6 h; 20% sucrose, 4–6 h; 30% sucrose, 12–36 h) containing 0.1% sodium azide. They were then embedded in Tissue-Tek OCT compound (Sakura Finetek Japan Co., Ltd, Tokyo, Japan). After instantaneously freezing, 10-μm-thick serial sections were prepared from the frozen blocks using a Tissue-Tek Polar instrument (Sakura Finetek Japan Co., Ltd). Each sample section was placed on an IR-reflective stainless-steel base and air dried for 24 h before measurement to prevent interference due of moisture. Serial sections were mounted onto glass slides, stained with using Periodic Acid Schiff (PAS) or immunofluorescent antibodies following standard methodology, and examined using a microscope to identify different microstructures.

### IR Microspectroscopy

*In situ* FT-IR measurements were performed using an IRT-7000 IR microscope combined with an FT/IR-6100 Fourier transform IR spectrometer (Jasco Co., Tokyo, Japan). The IR absorption spectra were acquired in reflection mode using a 16× Cassegrain lens and 30-μm × 30-μm apertures and collected in the mid-IR range of 700–4000 cm^-1^ at a resolution of 4 cm^-1^ over 64 scans. Reflection spectra were obtained from areas around blood vessels in the cerebral cortex using the lattice measurement method (*x*-axis: 7 points, *y*-axis: 7 points, total of 49 spectra acquired). Each spectrum was deconvoluted for protein secondary structural analysis. The averaged contents of the main protein conformations (α-helix, β-sheet, β-turn, and random coil) were estimated by measuring peak intensities around the amide I bands (1600–1700 cm^-1^), and were visualized using the universal RGB code on the protein mapping analysis software (IR-SSE; JASCO Co., Ltd) (Sarver and Krueger, 1991). Smoothing and normalization of spectra were performed on the region containing the amide bands (1000–2000 cm^-1^) using Spectra Manager software Ver. 2 (Jasco International Co., Ltd, Tokyo, Japan).

### PAS Staining and Immunofluorescence

Serial sections were stained using PAS or immunofluorescent antibodies (GFAP: Glial fibrillary acidic protein, MMR: Macrophage mannose receptor, and Aqp4: Aquaporin-4). PAS staining was performed to visualize PVM granules in the cerebral cortex. The sections were oxidized in 1% periodic acid solution for 1 min. After rinsing for 3 min in distilled water, the sections were soaked in cold Schiff reagent for 45 min. The sections were then soaked in sulfurous acid solution three times for 5 min and then rinsed for 3 min in distilled water. Finally, the sections were counterstained with hematoxylin for 1 s, then washed in flowing tap water and distilled water, dehydrated in graded alcohol, and cleared in xylene. Coverslips were applied using Permount mounting medium (Thermo Fisher Scientific Inc., Weltham, MA, USA).

Immunofluorescence was used to evaluate the protein expression patterns of Aqp4, GFAP, and MMR in the cerebral cortex. Sections were blocked in 10% normal donkey serum (IHR-8135, Immunobioscience, Mukilteo, WA, USA) for 1 h at room temperature and then incubated in primary goat polyclonal anti-GFAP antibody (code no. ab53554, Abcam, Cambridge, UK) diluted 1:500 in PBS for 16 h at 4°C. After rinsing three times for 5 min per rinse using PBS, the sections were further incubated in secondary Dylight 488-conjugated donkey anti-goat IgG (code no. 605-741-125, Rockland Immunochemicals Inc., Pottstown, PA, USA) diluted 1:1,000 in PBS for 120 min at room temperature and rinsed three times for 5 min per rinse with PBS. Sections were then incubated in primary rabbit polyclonal anti-Aqp4 antibody (code no. AB3594, Merck Millipore) diluted 1:200 in PBS or primary goat polyclonal anti-MMR/CD206 antibody (code no. AF2535, R&D Systems, Minneapolis, MN, USA) diluted 1:200 in PBS for 16 h at 4°C. After rinsing three times for 5 min per rinse with PBS, the sections were incubated in secondary Dylight 549-conjugated donkey anti-rabbit IgG (code no. 611-743-127, Rockland Immunochemicals, Inc.) diluted 1:1,000 in PBS or Dylight 549-conjugated donkey anti-goat IgG (code no. 605-742-002, Rockland Immunochemicals, Inc.) diluted 1:1,000 in PBS for 120 min at room temperature. The sections were then rinsed three times for 5 min per rinse with PBS and twice for 5 min per rinse with distilled water. Nuclei were counter-stained using Hoechst 33342 (code no. 346-07951, Dojindo Laboratories, Kumamoto, Japan).

### Statistical Analysis

All data are expressed as means ± standard deviations. Numbers and sex ratios of pups at birth and offspring body weights at 6 weeks of age were evaluated using unpaired *t*-tests. Proportions of secondary structure contents were analyzed using one-way analyses of variance followed by Tukey *post hoc* tests. The level of significance was set at *p* < 0.05. Statistical analyses were carried out using Excel Statistics 2012 (Social Survey Research Information, Tokyo, Japan).

## Results

### Litter Sizes and Offspring Body Weights

There were no significant between-groups differences in the numbers or sex ratios of offspring litters at birth (Supplementary Table 1) or offspring body weights at 6 weeks of age (Supplementary Table 2).

### Peak-Shift of Amide I Band in Perivascular Areas

**FIGURE 2:**
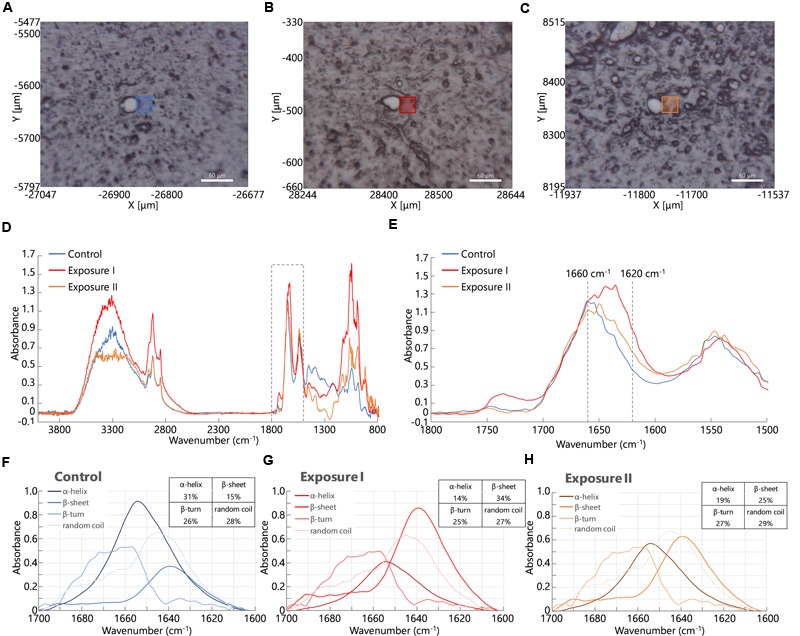
**Infrared microspectroscopy in perivascular areas.** Squares in **(A–C)** show brain areas where FT- IR spectra were measured in the control group **(A)**. Carbon black nanoparticles exposure group **(B,C)**. **(D)** Shows infrared spectra from each square (blue line: control group, orange and red lines: exposure group). **(E)** Shows enlarged spectra from the dashed line box in **(D)** to show the amide I peak-shift. **(F–H)** Spectra are obtained following deconvolution of the spectra in **(E)** using Spectra Manager software Ver. 2. The table at the upper right represents the proportions of the contents of each secondary structure calculated from peak intensities of the deconvoluted spectra around the amide I bands (Sarver and Krueger, 1991).

At first, we acquired FT-IR spectra from perivascular areas in the cerebral cortex from brain tissues in the control group (**Figure 2A**) and the maternal NPs exposure group (**Figures 2B,C**). **Figure 2D** shows the FT-IR spectra from each square (blue, orange, or red) in **Figures 2A–C**. The peak of the amide I band (approximately 1650 cm^-1^) was shifted by maternal NPs exposure. In contrast, the peak of the amide II band (approximately 1550 cm^-1^) was not shifted. We thus extracted the amide I band (**Figure 2E** is extracted from the dashed line box in **Figure 2D**, 1500–1800 cm^-1^) and analyzed protein secondary structures (**Figures 2F–H**). A broad peak at 1660 cm^-1^ and a shoulder at 1610–1640 cm^-1^ in the amide I region were seen in the spectrum of the control group (**Figure 2E**, blue line). The latter shoulder at 1610–1640 cm^-1^ of the spectra was enhanced in the maternal NPs exposure group compared to the control group (**Figure 2E**, orange and red lines). The former peak at 1660 cm^-1^ can generally be assigned to amide carbonyl bonds of α-helices in proteins, while the latter shoulder at 1610–1630 cm^-1^ represents the resonant frequency of the amide carbonyl group in β-sheet structures (Caine et al., 2012; Kawasaki et al., 2016a). Thus, the enhancement of the latter shoulder in the spectra obtained from the maternal NPs exposure group likely indicates an increase in β-sheet structure around blood vessels by maternal NPs exposure. To quantitatively examine the differences in the conformations of the proteins, each spectrum was deconvoluted using protein analysis software and calculated from the peak area of the deconvoluted spectra (**Figures 2F–H**) (Sarver and Krueger, 1991; Seshadri et al., 1999; Bousset et al., 2013). While the proportion of α-helix content was lower in the maternal NPs exposure group (**Figure 2G**: 14%, **Figure 2H**: 19%) than in the control group (**Figure 2F**: 31%), β-sheet content was higher in the NPs exposure group (**Figure 2G**: 34%, **Figure 2H**: 25%) than in the control group (**Figure 2F**: 15%). The proportions of β-turn and random coil content in the maternal NPs exposure group were almost the same as those in the control group.

Interestingly, two types of blood vessel were observed in one mouse in the maternal NPs exposure group: some had large peak-shifts (**Figure 2E**, red line), while others had small peak-shifts (**Figure 2E**, orange line) in the amide I region. Therefore, the calculated proportions of protein secondary structure were also different between these spectra in the maternal NPs exposure group. We wanted to determine the reason for the differences in the peak-shifts in the brain of one mouse in the exposure group. A previous study by our group indicates that low-dose maternal CB-NPs exposure induces diffuse perivascular abnormalities, including long-lasting astrogliosis and histopathological changes in PVMs (Onoda et al., 2014). We hypothesized that the differences in the peak-shifts of the amide I region in the brain of the mouse may be due to differences in histopathological denaturation around the blood vessels.

### Protein Secondary Structure Around Reactive Astrocytes

Glial fibrillary acidic protein, which is a marker of astrocyte activation and astrogliosis (Pekny and Nilsson, 2005), was dose-dependently upregulated by maternal CB-NPs exposure (Onoda et al., 2017). Increases in astrocytic GFAP expression frequently accompany increased Aqp4 expression (Haj-Yasein et al., 2011; Badaut et al., 2012). In agreement with the above evidence, Aqp4 was also dose-dependently upregulated by maternal CB-NPs exposure (Onoda et al., 2017). In particular, previous studies indicate that GFAP upregulation is remarkable in areas surrounding blood vessels and Aqp4 expression is further increased in GFAP-positive astrocytic end-feet in the brain parenchyma regions (>5 μm from vessels) than in the glia limitans region (proximal to blood vessels) by maternal CB-NPs exposure. Therefore, we examined secondary structural change in proteins at wider areas around the blood vessel where activated astrocytes have been observed or not.

**FIGURE 3:**
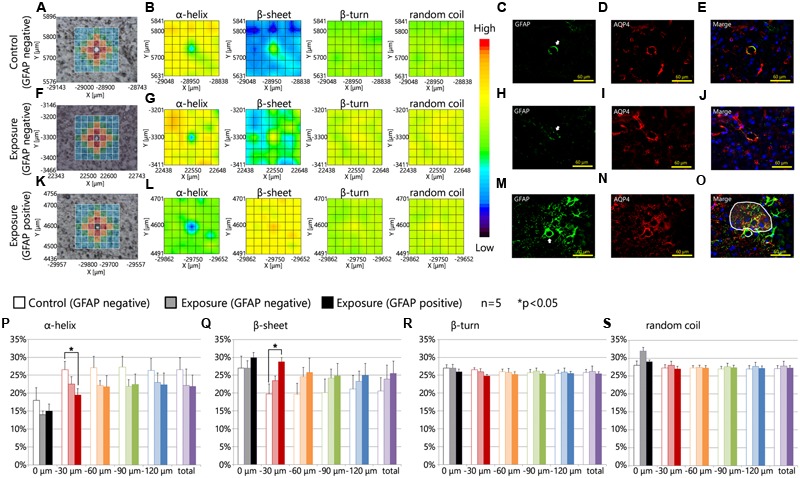
**Protein secondary structure around reactive astrocytes. (A,F,K)** Brightfield images showing the measured lattice (*x*-axis: 7 points, *y*-axis: 7 points, total of 49 spectra acquired). **(B,G,L)** 2D mapping images for each protein secondary structure (α-helix, β-sheet, β-turn, and random coil) obtained from the FT-IR spectra of the lattice. **(C–E,H–J,M–O)** Immunofluorescent photographs of each serial section from **(A,F,K)**, respectively. These figures show the GFAP (green) and aquaporin-4 (Aqp4, red) expression and nuclei (blue). White arrows in **(C,H,M)** indicate the centered blood vessels in **(A,F,K)**. White areas in **(O)** show the higher increase in Aqp4 expression in GFAP-positive astrocytic end-feet in the brain parenchyma (distal to the centered blood vessel) compared to those in the glia limitans region (proximal to the centered blood vessel) by maternal exposure to carbon black nanoparticles. **(P–S)** Represent the results of the quantitative and statistical analyses of the protein secondary structure around the blood vessel for each aperture at a different distance from the blood vessel at the center (0 μm: gray; 0–30 μm: red; 30–60 μm: orange; 60–90 μm: green; more than 90 μm: blue).

**Figures 3A–O** showed the results of the mapping analysis of the FT-IR spectra (**Figures 3A,B,F,G,K,L**) and the immunofluorescent staining (**Figures 3C–E,H–J,M–O**). These data were used to investigate the differences in the proportions of protein secondary structures between brain tissues around blood vessels with GFAP and Aqp4 up-regulation and tissues in areas without GFAP and Aqp4 increases. **Figures 3A,F,K** are bright-field images of the focused region in each serial section presented in **Figures 3C–E,H–J,M–O**, respectively. **Figures 3A,F,K** also showed the measured lattice. GFAP expression was upregulated around some blood vessels by maternal NPs exposure (**Figures 3M–O**). Aqp4 expression was also co-upregulated with GFAP expression at the end-feet of astrocytes in the brain parenchyma regions by maternal NPs exposure (**Figures 3M–O**), in agreement with previous studies. 2D mapping of protein secondary structures (**Figures 3B,G,L**) indicated that the β-sheet content was more increased and the α-helix content was more decreased around blood vessels with the co-upregulation of GFAP and Aqp4 (**Figure 3L**) than around blood vessels without GFAP and Aqp4 over-expression in the maternal NPs exposure group (**Figure 3G**). Our results thus indicate that the peak-shift of the amide I region is related to the astrogliosis induced by maternal CB-NPs exposure. The proportions of the β-turn and random coil contents were not altered in the areas surrounding blood vessels regardless of the presence or absence of histopathological changes in astrocytes (**Figures 3B,G,L**).

Furthermore, the effects of maternal NPs exposure were clearly seen in the protein secondary structural analysis for each aperture at different distances from blood vessels (**Figures 3A,F,K,P–S**; 0 μm: gray; 0–30 μm: red; 30–60 μm: orange; 60–90 μm: green; more than 90 μm: blue). To investigate the proportions of protein secondary structures around the blood vessels quantitatively and statistically at each aperture, the spectra from all apertures in the lattice were deconvoluted and the proportions of each conformation were calculated using the same methods as those described in **Figure 2**. The proportion of the β-sheet content was significantly increased in areas close mostly to the centered blood vessel in each measured area in the lattice (red area: 0–30 μm from the centered blood vessel) containing astrocytes with high expression levels of GFAP in the maternal CB-NPs exposure group (^∗∗^*p* < 0.01) compared to areas around blood vessels without GFAP increases in the control group (**Figure 3Q**). In contrast, the α-helix content around blood vessels was significantly decreased by maternal exposure to CB-NPs (^∗∗^*p* < 0.01) compared to areas around blood vessels without GFAP increases in the control group (**Figure 3P**). The proportion of each content was not significantly altered in areas around blood vessels without GFAP and Aqp4 co-upregulation in the maternal CB-NPs exposure group compared to the control group (**Figures 3P–S**).

**Table 1** shows the amide I peak wavenumbers in four areas close mostly to the centered blood vessel in each measured area in the lattice (red squares). While the peak wavenumbers ranged from 1651 to 1661 cm^-1^ in the control group, they ranged from 1648 to 1657 cm^-1^ at blood vessels without GFAP over-expression and from 1636 to 1654 cm^-1^ at blood vessels with GFAP up-regulation in the CB-NPs exposure group. The shift of the amide I peak from a high wavenumber to a low wavenumber supports the results that increases in β-sheet content are related to GFAP up-regulation induced by maternal NPs exposure.

**Table 1:** Amide I peak wavenumbers in four squares close mostly to the centered blood vessel in the lattice (red squares in **Figure 3**).

Group name	Mouse ID	Wave-number (cm^-1^)	Means ± standard deviations
Control	C1	1655.6	1655.4 ± 3.2
		1651.7	
		1659.5	
		1654.6	
	
	C2	1654.6	1652.0 ± 4.1
		1646.0	
		1654.6	
		1652.7	
	
	C3	1657.5	1658.8 ± 1.0
		1659.5	
		1659.5	
		1658.5	
	
	C4	1660.4	1660.9 ± 0.6
		1660.4	
		1661.4	
		1661.4	
	
	C5	1657.5	1657.0 ± 1.7
		1658.5	
		1657.5	
		1654.6	

Exposure GFAP	E1	1654.6	1652.2 ± 2.9
negative sites		1648.8	
		1654.6	
		1650.8	
	
	E2	1655.6	1654.2 ± 3.3
		1657.5	
		1649.8	
		1653.7	
	
	E3	1651.7	1653.2 ± 1.3
		1652.7	
		1654.6	
		1653.7	
	
	E4	1649.8	1652.7 ± 2.1
		1652.7	
		1653.7	
		1654.6	
	
	E5	1648.8	1653.9 ± 3.5
		1656.6	
		1655.6	
		1654.6	

Exposure GFAP	E1	1648.8	1651.0 ± 2.8
positive sites		1654.6	
		1651.7	
		1648.8	
	
	E2	1636.3	1642.6 ± 6.8
		1646.9	
		1649.8	
		1637.3	
	
	E3	1651.7	1648.4 ± 2.4
		1646.0	
		1647.9	
		1647.9	
	
	E4	1646.0	1649.3 ± 2.8
		1652.7	
		1649.8	
		1648.8	
	
	E5	1651.7	1648.6 ± 3.8
		1651.7	
		1644.0	
		1646.9	

*The table shows the wavenumbers of the amide I peaks for four squares close mostly to the centered blood vessel in each measured area in the lattice (red squares in **Figure 3**). In the control group, the data for four squares around one blood vessel in each mouse (*n* = 5 per group) are shown. In the carbon black nanoparticles exposure group, the data for a blood vessel with GFAP-negative astrocytes and another blood vessel with GFAP-positive astrocytes in each mouse are presented.*

### Relationship between Changes in Protein Secondary Structure and Perivascular Macrophages

Previous studies have also shown that low-dose maternal CB-NPs exposure induces diffuse histopathological changes in PVMs (Onoda et al., 2014). We thus performed mapping analysis of FT-IR spectra on brain tissues around blood vessels with PVMs. We performed double immunofluorescence experiments probing for GFAP and MMR (CD206), which is a specific marker of PVMs (Galea et al., 2005). We also carried out PAS staining to detect the denaturation of lysosomal granules in PVMs (Mato and Ookawara, 1979; Mato et al., 2002).

**FIGURE 4:**
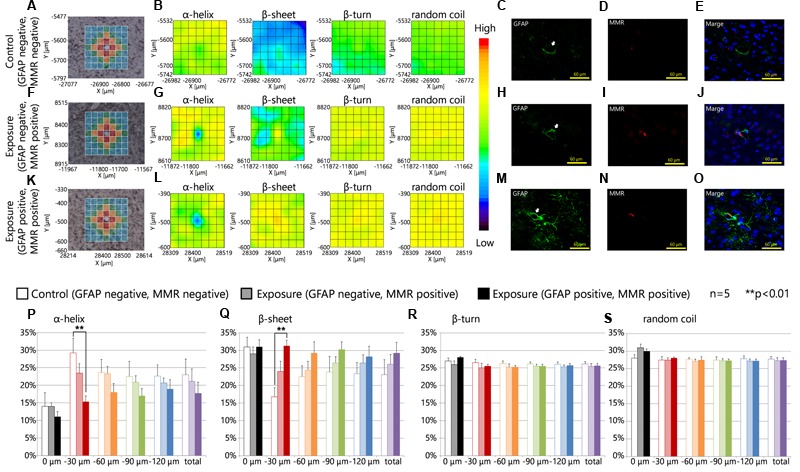
**Protein secondary structure and perivascular macrophages. (A,F,K)** Bright-field images showing the measured lattice (*x*-axis: 7 points, *y*-axis: 7 points, total of 49 spectra acquired). **(B,G,L)** 2D mapping images for each protein secondary structure (α-helix, β-sheet, β-turn, and random coil) obtained from the FT-IR spectra for the lattice. **(C–E,H–J,M–O)** Immunofluorescent photographs of each serial section from **(A,F,K)**, respectively, and show the GFAP (green), and macrophage mannose receptor (MMR, red) expression, and nuclei (blue). White arrows indicate the centered blood vessel in **(A,F,K)**. **(P–S)** Represent the results of the quantitative and statistical analyses for protein secondary structure around the blood vessel for each aperture at different distances from the blood vessel at the center (0 μm: gray; 0–30 μm: red; 30–60 μm: orange; 60–90 μm: green; more than 90 μm: blue).

**Figures 4A,F,K** are brightfield images of the focused region in each serial section presented in **Figures 4C–E,H–J,M–O**, respectively, and show the measured lattice. The expression of MMR was not related to the expression levels of GFAP in the control group or the maternal NPs exposure group (**Figures 4C–E,H–J,M–O**). 2D mapping images of protein secondary structures (**Figures 4B,G,L**) indicated that the β-sheet content was increased and the α-helix content was decreased around blood vessels in the maternal NPs exposure group (**Figures 4G,L**) compared to the control group (**Figure 4B**). The large increase in β-sheet content and the large decrease in α-helix content were only observed around blood vessels with MMR-positive cells (PVMs) and GFAP upregulation (**Figure 4L**), but not around blood vessels with PVMs without GFAP over-expression (**Figure 4G**). These results indicate that the existence of PVMs is not crucial for the peak-shift of the amide I region, which is correlated to alterations in the proportions of protein secondary structure. The proportions of the β-turn and random coil contents in each area around the blood vessel were not altered regardless of the presence of PVMs and the upregulation of GFAP (**Figures 4B,G,L**).

The effects of maternal NPs exposure were clearly reflected in the results of the protein secondary structure analysis at each aperture at different distances from the blood vessel at the center (**Figures 3A,F,K,P–S**, 0 μm: gray; 0–30 μm: red; 30–60 μm: orange; 60–90 μm: green; more than 90 μm: blue). The proportion of the β-sheet content was significantly increased in areas close mostly to the centered blood vessel in each measured area in the lattice (red area: 0–30 μm from the centered blood vessel) with PVMs and astrocytes with high expression levels of GFAP in the maternal CB-NPs exposure group (^∗∗^*p* < 0.01) compared to the control group (**Figure 4Q**). In addition, the α-helix content around the blood vessels was significantly decreased in areas close mostly to the centered blood vessel in each area of the lattice (red area: 0–30 μm from the centered blood vessel) with PVMs and astrocytes with high expression levels of GFAP in the maternal CB-NPs exposure group (^∗∗^*p* < 0.01) compared to the control group (**Figure 4P**). In contrast, the proportions of β-sheet and α-helix contents were not significantly altered in areas around blood vessels with PVMs without GFAP over-expression compared to the control group (**Figures 4P,Q**). These results supported the results that the existence of PVMs is not related to the peak-shift in the amide I region, which is correlated with alterations in protein secondary structure.

Although the existence of PVMs did not contribute to the peak-shift of the amide I region by maternal CB-NPs exposure, there is still the possibility that the denaturation of PVMs relates to the peak-shift. This is because maternal CB-NPs induces histopathological changes in PVMs with enlarged lysosomal granules, which is a typical histopathological finding following denaturation of PVMs (Onoda et al., 2014). Therefore, we acquired FT-IR spectra at wider areas surrounding blood vessels with and without enlargement of lysosomal granules in PVMs.

**FIGURE 5:**
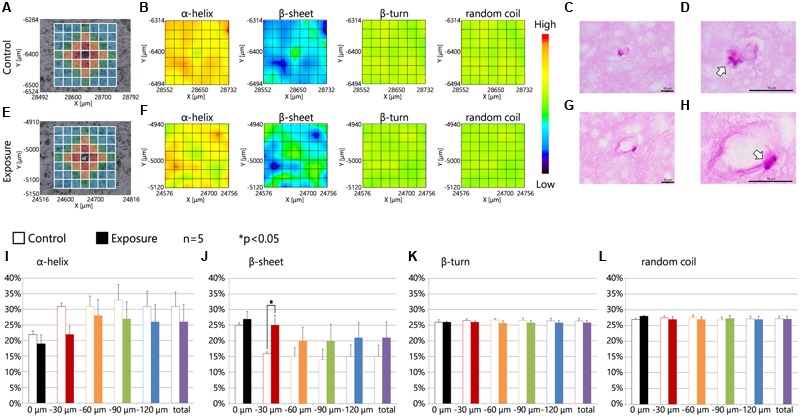
**Protein secondary structure around perivascular macrophages with enlarged granules. (A,E)** Brightfield images showing the measured lattice (*x*-axis: 7 points, *y*-axis: 7 points, total of 49 spectra acquired). **(B,F)** 2D mapping images of each protein secondary structure (α-helix, β-sheet, β-turn, and random coil) obtained from the FT-IR spectra for the lattice. **(C,D,G,H)** Periodic acid-Schiff (PAS) staining photographs for each serial section in **(A,E)** respectively. **(D,H)** Enlarged photographs of **(C,G)** and show the lysosomal granules in perivascular macrophages (arrows). **(I–L)** Represent the results of the quantitative and statistical analyses for the protein secondary structure around the blood vessel for each aperture at different distances from the blood vessel at the center (0 μm: gray; 0–30 μm: red; 30–60 μm: orange; 60–90 μm: green; more than 90 μm: blue).

Intracellular lysosomal granules, which are a feature of PVMs, were stained red with PAS (**Figures 5C,G**). The photographs showed that the PAS-positive granules were enlarged by maternal NPs exposure when compared to lysosomal granules in PVMs in the control group (**Figures 5D,H**, arrows). **Figures 5A,E** show sections in series with those shown in **Figures 5C,G** and show the measured lattice. 2D mapping data of protein secondary structures showed that maternal CB-NPs exposure induces an increase in β-sheet content and a decrease in α-helix content around blood vessels with enlarged lysosome granules in PVMs in the maternal NPs exposure group (**Figure 5F**) compared to blood vessels without denatured granules in PVMs in the control group (**Figure 5B**). Although the presence or absence of MMR-positive cells (PVMs) did not affect the alterations in secondary structure proportions induced by maternal NPs exposure (**Figure 4**), the denaturation of lysosomal granules in PVMs was related to the peak-shift of the amide I region by maternal NPs exposure. In particular, the proportion of the β-sheet content was significantly increased in areas close mostly to the centered blood vessel in each measured area in the lattice (red area: 30 μm from the blood vessel) with enlarged lysosomal granules in the maternal CB-NPs exposure group compared to the control group (**Figure 5J**). The proportions of α-helix, β-turn, and random coil contents were not altered regardless of the presence or absence of PVMs with denatured granules (**Figures 5I,K,L**).

## Discussion

Previous studies have demonstrated that maternal NPs exposure induces long-lasting diffuse perivascular abnormalities, including astrogliosis and histopathological changes in PVMs in offspring mice (Onoda et al., 2014, 2017). The purpose of the present study was to widely investigate histopathological denaturation in the neurovascular areas during brain development. In particular, we expected to be quantitatively and comprehensively examine the chemical and structural compositions of biomolecules using *in situ* FT-IR on biological tissue sections. Our results indicate that the peak of the amide I band region in FT-IR spectra in perivascular areas was shifted by maternal NPs exposure. However, there were two types of peak-shift in one mouse in the exposure group: some areas had a large peak-shift and others had a small peak-shift. Large peak-shifts of the amide I band in particular resembled those seen in regions of amyloid-β accumulation in AD tissue (Choo et al., 1996). Using *in situ* FT-IR in combination with traditional staining methods, we found that the large peak-shifts were induced around blood vessels with perivascular abnormalities with astrocytes with high GFAP and Aqp4 expression levels and enlarged lysosome granules in PVMs in the brains of offspring mice from the maternal NPs exposure group. Furthermore, protein secondary structural analysis indicated that maternal NPs exposure increases β-sheet content and decreases α-helix content in areas close mostly to the centered blood vessel with histopathological changes. As far as we are aware, this is the first study on the changes in protein secondary structure at various distances from blood vessels with histopathological changes in the brain. These data indicate that β-sheet-rich protein accumulates in regions with perivascular abnormalities, such as astrogliosis and denaturation of PVMs, induced by maternal NPs exposure.

In this study, we focused on the amide I band (1600–1700 cm^-1^) because the peak of the amide I band was most drastically shifted in the FT-IR spectra, while the peak of the amide II band was not shifted. In fact, the peak of the amide I band sensitively reflects alterations in secondary protein structure (Sekar et al., 2015). Circular dichroism (CD) spectroscopy is another excellent tool used to estimate the secondary structures of proteins (Greenfield, 2006). However, CD spectroscopy cannot provide information regarding the locations of the alterations in secondary protein structure on tissue sections because CD spectra are measured only in aqueous solutions (Pelton and McLean, 2000). Because the purpose of the present study was to quantitatively and comprehensively investigate the biochemical and structural alterations in biomolecules around blood vessels and to elucidate the relationships between the alterations in these biomolecules and histopathological changes in brain tissue, *in situ* FT-IR was the most suitable technique for use in the present study. The minimum unit in the lattice used in the *in situ* FTIR was 30-μm square in area. FT-IR spectra obtained in areas smaller than 30-μm square is unstable and unreliable due to insufficient light, and in this case, led to failure in calculating protein secondary structure from the spectra. Therefore, the aperture used for the FT-IR spectra in the present study was set to 30-μm square in order to achieve the maximum mapping resolution and reliable analysis of protein secondary structure. Analysis of protein secondary structure was performed using software referred to in a study by Sarver and Krueger (1991). This study described the methods to calculate protein secondary structure by investigating the relationships between the peaks of the amide I band in the FT-IR spectra and secondary structures of 17 types of proteins whose conformations had been completely revealed by X-ray crystal structure analysis. This study also showed that FT-IR is slightly more sensitive than CD for estimating the amount β-sheet content. Therefore, the *in situ* FT-IR technique used in the present study is the best approach for the examination of histopathological denaturation in wide neurovascular areas in the brain tissues.

The surface reactivity of NPs is important for understanding the alterations in protein secondary structures by maternal NPs exposure. Many previous studies have shown that biomolecules, including proteins and lipids, adsorb onto the surfaces of NPs and form biomolecular/protein corona by enshrouding the NPs in biological environments (Saptarshi et al., 2013; Treuel et al., 2015). This reaction on the surface of NPs sometimes induces conformational changes in the adsorbed protein (Saptarshi et al., 2013). Protein secondary structure is especially sensitive to interactions with NPs (Wang et al., 2011; Khanal et al., 2016). Eventually, proteins with altered conformations are eliminated from the central nervous system as waste proteins. Alterations in proportions of protein secondary structure by maternal NPs exposure may be due to interactions between the surface of the NPs and proteins in the brains of offspring. In fact, NPs with diameters of 1–100 nm are translocated to the fetal brain (Takeda et al., 2009; Yamashita et al., 2011), as they pass through biological barriers, such as the blood-air barrier in the lung of the dam (Kreyling et al., 2002; Oberdörster et al., 2002; Choi et al., 2010) and the blood-placental barrier between the mother and the fetus (Wick et al., 2010). The previous study have confirmed the transplacental transfer of NPs with diameters of 240 nm or less by using *ex vivo* perfusion model of healthy human placenta. Moreover, NPs can be detected in the brains of offspring mice up to 6 weeks after birth as determined by elemental analysis using scanning electron microscopy/energy-dispersive X-ray spectroscopy in the case of titanium dioxide (Takeda et al., 2009). When carbon black particles injected into the cerebral ventricle, they have also stayed in the brain cells for more than 2 years in rat models (Kida et al., 1993). These data have indicated that it is likely difficult to excrete and/or dispose of NPs from the brain and that biomolecules may interact with the surface of the NPs over a long period of time. Therefore, maternally administered NPs likely translocate from the dam to the offspring brain, react with proteins, and induce protein conformational changes leading to increasing β-sheet content and decreasing α-helix content. Moreover, fetal brain dramatically develops by constructing a neural network with glial cells and blood vessels (Kolb and Gibb, 2011). Foreign substances like NPs may induce irreversible changes in the network by interaction with the various developing cells and biomolecules (Calderón-Garcidueñas et al., 2016a). The impact of NP exposure during pregnancy is likely greater than the exposure in adults.

Neuroinflammation during pregnancy should also be discussed when considering alterations in secondary protein structure. Previous studies have clearly demonstrated that infection during pregnancy induces neuroinflammation in the fetal and neonatal brain, and that inflammation in the brain has adverse effects on brain development (Atladóttir et al., 2010; Brown, 2012). Emerging evidence also suggests that maternal exposure to particulate air pollutants induces neuroinflammation in the fetal brain (Bolton et al., 2012; Hougaard et al., 2015). Importantly, neuroinflammation during development leads to the misfolding of proteins (Soto, 2003; Amor et al., 2010) and promotes the generation and accumulation of various types of waste proteins composed of β-sheet-rich structures (Stefani, 2004). Therefore, it may also be important to consider the neuroinflammation induced by maternal NPs exposure when studying alterations in the proportions of secondary protein structures.

The present study showed the changes in β-sheet content were remarkable in areas close mostly to the centered blood vessel. More β-sheet-rich proteins are likely to accumulate for discharge to perivascular space, which is one of the clearance routes for brain waste (Iliff et al., 2012; Xie et al., 2013). In the CNS, interstitial fluids constantly flow into the brain parenchyma and out of the perivascular space (Virchow-Robin space) with waste products through Aqp4 water channels (Iliff et al., 2012; Kress et al., 2014). In other words, waste in the brain, including proteins with changed conformations, is removed and accumulates in the perivascular space due to the flow of interstitial fluid via Aqp4 channels. Brain waste proteins are also eliminated by smooth muscle cells surrounding blood vessels (Weller et al., 2008). In any case, waste proteins, which are unnecessary in the brain, are removed to the perivascular space. Thus, the increase in β-sheet content in the areas, close mostly to blood vessels, likely indicates the accumulation of waste proteins denatured by maternal NPs exposure in the perivascular space by these clearance systems in the brain. In addition, we hypothesized that the translocated NPs accumulated around blood vessels and were reactive against biomolecules. However, previous studies have failed to detect CB-NPs using transmission electron microscopy either inside or outside cells surrounding blood vessels, including PVMs or astrocytes, in offspring mice from mothers intranasally exposed to CB-NPs (95 μg/kg) (Onoda et al., 2014). Therefore, it is likely that NPs induce the conformational denaturation of proteins in the brain parenchyma regions, but not in perivascular regions.

*In situ* FT-IR revealed that β-sheet-rich proteins accumulated in regions with perivascular abnormalities, such as astrogliosis and denatured PVMs, induced by maternal NPs exposure. These observations may be strongly connected to the above clearance systems for waste proteins. The accumulated waste proteins in the perivascular areas are usually phagocytosed by PVMs, which exist and migrate in the perivascular space surrounded by glia limitans and vascular endothelial cells (Kida et al., 1993; Lai and McLaurin, 2012; Serrats et al., 2010). Because continued phagocytosis induces the denaturation and enlargement of PAS-positive lysosomal granules in PVMs (Mato et al., 2002, 2009), the denaturation of PVMs by maternal NPs exposure may be due to the excess phagocytosis of β-sheet-rich proteins, which are likely to be removed as waste proteins. Furthermore, the denaturation of PVMs is related to GFAP over-expression in astrocytes. Previous studies have demonstrated that GFAP up-regulation by maternal NPs exposure is observed around blood vessels with enlarged lysosomal granules in the PVMs (Onoda et al., 2014). In the present study, β-sheet content was also increased around blood vessels with GFAP and Aqp4 up-regulation, as well as around blood vessels with denatured PVMs. Because GFAP and Aqp4 are markers of typical astrocyte activation (Pekny and Nilsson, 2005; Haj-Yasein et al., 2011; Badaut et al., 2012), these results suggest that β-sheet-rich proteins accumulate around persistently activated astrocytes. Alternatively, the astrocyte activation may be induced by the β-sheet-rich proteins accumulated in the perivascular space and/or by PVMs denatured by the excessive phagocytosis of β-sheet-rich proteins. In particular, Aqp4 has a key role in the flow of interstitial fluids involved in the waste protein clearance system (Kress et al., 2014). Astrocytes are activated and highly express Aqp4 when they eliminate β-sheet-rich proteins, which are likely to be removed as waste proteins (Vajda et al., 2000; Papadopoulos and Verkman, 2007). Therefore, GFAP and Aqp4 upregulation and denaturation of PVMs induced by maternal NPs exposure is likely caused by the accumulation of waste proteins around blood vessels, as first determined using a combination of *in situ* FT-IR and traditional staining methods in the present study. Furthermore, the accumulation of β-sheet content around blood vessels with histopathological changes in PVMs and astrocytes suggests that the elimination of these proteins by the clearance systems in the brain may not be able to keep up with the generation of β-sheet-rich proteins by maternal NPs exposure.

Protein conformational changes are caused by inflammation (Schain and Kreisl, 2017), aging (Chauhan, 2014; Van Raamsdonk et al., 2017), traumatic injury (Chauhan, 2014), and infection (Perry et al., 2003; Rock et al., 2004) in the CNS. These causes of the protein conformational changes in the brain also induce the blood–brain barrier disruption concomitant with dysregulation of Aqp4 expression (Haj-Yasein et al., 2011; Erdö et al., 2017; Schain and Kreisl, 2017). Because the conformational denaturation of proteins was observed around brain perivascular abnormalities with Aqp4 upregulation in astrocytes, maternal CB-NP exposure may also affect blood–brain barrier function. The histopathological changes of astrocytes and PVMs following maternal CB-NP exposure may be associated with the blood–brain barrier disruption. In addition, the alteration of Aqp4 expression levels following maternal CB-NP exposure may induce the dysregulation of water balance in the CNS. In fact, previous studies with ultrastructural pathological analysis by electron microscopy showed that the maternal CB-NP exposure causes severe swelling of astrocytic end-feet with denatured PVMs in the brain of offspring (Onoda et al., 2014). The water imbalance is related to dysregulation of ionic, cholinergic, and nitric oxide metabolism which probably cause CNS disease (Liu et al., 2012; Mack and Wolburg, 2013; Oku et al., 2015). In particular, because the changes in expression profiles of GFAP and Aqp4 in offspring after maternal CB-NP exposure were similar to those in observed in mice of advanced age (Onoda et al., 2017), the cholinergic manipulation may be also suppressed as observed in aged subjects (Schliebs and Arendt, 2011). Evaluation of blood–brain barrier disruption, detailed water imbalance, and cholinergic neuron function is also an important issue and requires further study. Potential epigenetic change is of interest to understand the mechanism of long-lasting GFAP and Aqp4 up-regulation.

Finally, the clinical implications of β-sheet-rich protein accumulation around blood vessels with perivascular abnormalities and the development of neurodegenerative disease merits discussion. Clinical evidence suggests that prenatal exposure to particulate air pollutants leads to diffuse damage to the neurovascular unit in the developing brain and predisposes individuals to neurodegenerative diseases, including AD and PD (Calderón-Garcidueñas et al., 2013, 2016a,b; Heusinkveld et al., 2016). Neurodegenerative diseases are included in conformational diseases induced by the deposition of proteins with abnormal structures (Zerovnik, 2002; Gidalevitz et al., 2010). Abnormal protein structure often leads to aberrant protein-membrane interactions, mislocalization, degradation, and aggregation, ultimately resulting in neurodegenerative disease due to decreased availability of functional proteins and increases in the proteins with newly obtained toxic function (Bucciantini et al., 2002; Gidalevitz et al., 2010). In fact, deposition of abnormally structured proteins is observed in almost all neurodegenerative diseases, including AD and PD (Soto and Estrada, 2008). Therefore, abnormally structured proteins must be sufficiently eliminated as waste by clearance systems in order to maintain the normal state and function of the brain. In particular, waste proteins that cause conformational diseases due to structural changes are naturally β-sheet-rich or convert to β-sheet-rich structures from their native conformations (Zerovnik, 2002; Meredith, 2005). The conversion to β-sheet-rich structures induces cytotoxicity of the protein (Bucciantini et al., 2002). FT-IR studies have in fact revealed that waste proteins, such as amyloid-β and proteins comprising Lewy bodies, which are neuropathological hallmarks of AD and PD, respectively, are mainly composed of β-sheets (Choo et al., 1996; Araki et al., 2015). In Huntington disease, conformation of huntingtin protein is changed into β-sheet-rich structures that aggregate into potentially toxic oligomeric species and fibril structures (Smaoui et al., 2016). The secondary structure of prion proteins that induce the severe conformational diseases of the brain, Creutzfeldt-Jakob disease and mad cow disease, clearly changes from α-helix to β-sheet-rich (Ronga et al., 2007; Baskakov, 2009; Coleman et al., 2009). Thus, β-sheet-rich proteins may be key factors in the development of neurodegenerative disease. The published evidence and the data from the present study suggest that the risk of the development of neurodegenerative diseases may be enhanced following exposure to particulate air pollutants during brain development and that this may be due to increases in and accumulation of β-sheet-rich proteins. Change in expression patterns of GFAP and Aqp4 in astrocytes around blood vessels and the increase in β-sheet content in the maternal NPs exposure group was particularly similar to the histopathological changes observed in brains with local neurodegeneration (Maragakis and Rothstein, 2006; Foglio and Rodella, 2010). Therefore, using *in situ* FT-IR in combination with traditional staining methods, we likely captured the pathological conditions present during the early stages of neurodegeneration. This is essential for understanding the acceleration of diseases induced by exposure to particulate air pollutants during brain development.

## Conclusion

Here we report that the peak of the amide I band region in FT-IR spectra obtained from perivascular areas shifts from a high wavenumber to a low wavenumber by maternal NPs exposure. The peak-shift was remarkable around perivascular abnormalities with over-expression of GFAP and Aqp4 in astrocytes and enlarged lysosome granules in PVMs in brains of offspring mice, as detected using a combination of *in situ* FT-IR and traditional staining methods. Protein secondary structural analysis at each distance from the centered blood vessel in the brain indicated increases in β-sheet content and decreases in α-helix content in areas mostly close to the centered blood vessel with histopathological changes. These results suggest that β-sheet-rich waste proteins, which are denatured by maternal NPs exposure, likely accumulate in the perivascular space due to clearance systems in the brain and lead to astrocyte activation and PVM denaturation. β-sheet-rich proteins may be key factor in the induction of neurodegeneration by maternal exposure to particulate air pollutants. The present data will contribute to future investigations examining the features and mechanisms of pathological changes in neurodegenerative diseases induced by various environmental risk factors during brain development.

## Availability of Data and Materials

Data supporting the findings are found within the manuscript. Raw data files will be provided by the corresponding author upon request.

## Author Contributions

AO mainly performed all experiment procedures and data analyses, and drafted the manuscript. TK substantially involved in analysis with FT-IR microspectroscopy and drafting manuscript. KoT substantially involved in organizing and managing the FT-IR analyses. KeT is main project leader and conceived the idea of research on developmental effects of NPs. MU is co-leader of this project, conceived the overall research idea and involved in drafting manuscript. All authors have read and approved the final manuscript.

## Conflict of Interest Statement

The authors declare that the research was conducted in the absence of any commercial or financial relationships that could be construed as a potential conflict of interest.
