# Supplementary material for: Perivascular Accumulation of β-Sheet-Rich Proteins in Offspring Brain following Maternal Exposure to Carbon Black Nanoparticles
Source: Front Cell Neurosci. 2017 Mar 31;11:92. doi: 10.3389/fncel.2017.00092 (PMC5374146; doi:10.3389/fncel.2017.00092)
Supplement: Supplementary file 1 [file Table_1.DOCX]

Supplementary Table 1. Effects of maternal exposure to carbon black nanoparticles on litter size and offspring sex ratio

| **Group name** | **Number of dams** | **Number of offspring** | **Sex ratio (%)*** |
| --- | --- | --- | --- |
| Control | 5 | 19 ± 2 | 52 ± 14 |
| CB-NPs | 5 | 17 ± 3 | 50 ± 16 |

There were no significant between-groups differences. Data are presented as means ± standard deviations. *Sex ratio (%) = male/(male + female) × 100. Abbreviations: CB-NPs, carbon black nanoparticles
