# Supplementary material for: Perivascular Accumulation of β-Sheet-Rich Proteins in Offspring Brain following Maternal Exposure to Carbon Black Nanoparticles
Source: Front Cell Neurosci. 2017 Mar 31;11:92. doi: 10.3389/fncel.2017.00092 (PMC5374146; doi:10.3389/fncel.2017.00092)
Supplement: Supplementary file 2 [file Table_2.DOCX]

Supplementary Table 2. Effect of maternal exposure to carbon black nanoparticles on male offspring body weight

| **Group name** | **Number of dams**  **(Number of offspring)** | **Age** | **Body weight (g)** |
| --- | --- | --- | --- |
| Control | 5 (20) | 6 weeks | 36 ± 4 |
| CB-NPs | 5 (20) | 6 weeks | 35 ± 2 |

There were no significant between-groups differences. Data are presented as means ± standard deviations. Abbreviations: CB-NPs, carbon black nanoparticles
